# Different Effects of Regional Species Pool on Plant Diversity between Forest and Grassland Biomes in Arid Northwest China

**DOI:** 10.1371/journal.pone.0131982

**Published:** 2015-07-02

**Authors:** Liping Li, Yining Liu, Xiangping Wang, Jingyun Fang, Qingchun Wang, Bengang Zhang, Peigen Xiao, Anwar Mohammat, André Terwei

**Affiliations:** 1 Key Laboratory of Bioactive Substances and Resources Utilization of Chinese Herbal Medicine (Peking Union Medical College), Ministry of Education, Institute of Medicinal Plant Development, Chinese Academy of Medical Sciences, Peking Union Medical College, Beijing, China; 2 Department of Ecology, College of Urban and Environmental Sciences, Key Laboratory for Earth Surface Processes of the Ministry of Education, Peking University, Beijing, China; 3 The Key Laboratory of Silviculture and Conservation of the Ministry of Education, College of Forestry, Beijing Forestry University, Beijing, China; 4 College of Nature Conservation, Beijing Forestry University, Beijing, China; 5 Xinjiang Institute of Chinese and Ethnic Medicine, Urumqi, China; 6 Xinjiang Institute of Ecology and Geography, Chinese Academy of Sciences, Urumqi, China; 7 Department for Ecological Interactions, German Federal Institute of Hydrology, Koblenz, Germany; Chinese Academy of Forestry, CHINA

## Abstract

Species pool hypothesis is broadly known and frequently tested in various regions and vegetation types. However it has not been tested in the arid Xinjiang region of China due to lack of data. Here with systematic data from references and field survey, we comprehensively examined species pool hypothesis in this region. Took species richness in 0.1° × 0.1° grid cells as regional species richness (RSR) which were obtained from the distribution maps of vascular plant species, and took species diversity of 190 and 103 plots in forest and grassland biomes across Xinjiang as local species richness (LSR), together with the digitalized soil pH and climate data, we tested the species pool hypothesis in this region. We found that: (1) the average RSR was higher in mountains than that in basins and it was negatively correlated with soil pH in mountains while positively correlated with soil pH in basins in Xinjiang; (2) RSR showed a positive correlation with mean annual precipitation (MAP) while showed a hump-shaped pattern with mean annual temperature (MAT); and the changing patterns of LSR were different for forest and grassland along the geographical and climate gradients; (3) LSR of forest was more affected by RSR than by climate, while on the contrary, LSR of grassland was more affected by climate than by RSR. Our results validated the species pool hypothesis in revealing that RSR had a significant role in shaping LSR patterns in addition to climate. We concluded that the relative effects of climate vs. RSR on LSR differed markedly between the forest and grassland communities across Xinjiang. Our results also showed that RSR revealed a contrasting relationship with soil pH in mountains and in basins, which might reflect differences in evolutionary processes of various habitats. In summary, our research systematically analyzed the correlation of species richness in regional and local scales in Xinjiang which provides more insights into the understanding of species pool hypothesis.

## Introduction

Understanding biodiversity patterns is one of the central tasks of ecology [1]. Traditional biodiversity hypotheses explain most of the species richness patterns with local processes, for example, interspecific competition, abiotic filtering, and climatic variables [2,3]. Unfortunately, local processes could not fully explain biodiversity patterns in some regions or vegetation types, e.g., the species richness is higher in East Asia than in North America and Africa with similar environments [4,5], and the relationship of species richness—productivity in forest and grassland is different [6]. Thus, the potential effects of regional processes [7,8] and historical processes [9] were gradually recognized.

Species pool hypothesis is an alternative explanation for species distributions [10]. It was preliminarily proposed and improved in the 1990s [8,10–14], and more research was conducted in the 2000s and later [1,7,15,16]. It takes regional processes into account when explaining species richness patterns which were formerly neglected. According to the definition, the regional species pool, or regional species richness (RSR), is the set of species that is capable of coexisting in a community in a certain region; the local species pool, or local species richness (LSR), is species that actually presents in the community [1]. Species pool hypothesis does not exclude the effect of local ecological processes, such as competition and predation [8]. It holds that regional and local processes jointly control the plant community structure and species composition and the relative effects of the two might differ in various environments [1]. Till now how and in which extent regional processes shape the local ecological communities have not been fully understood [17]. Furthermore, the widely used regression method of testing the local and regional species richness was questioned because of the not independent relationships of the two [18].

Soil pH reflects the historical processes which may influence the speciation process [1]. Species richness would be positively correlated with soil pH in high latitude if it is influenced by regional species pool and vice versa in low latitude [19]. Different relationships of species richness and soil pH were found in various vegetation types in small scales, e.g., tundra, steppe and forest in Siberia [20], or forest, grassland, mire and sand in Central Europe [21]. How the relationship of RSR and pH in arid region is has not been tested yet.

Test of regional effect needs both regional and local species richness data; thus data limitations remain perhaps the most formidable obstacles to research on this topic [17]. Most empirical studies evaluated only the influence of either local environmental variables or regional enrichment but not simultaneously both. The most common method used to get the data of regional species pool is to apply Ellenberg indicator values which in fact are not easy to obtain [14]. In this research, we got the potential species distribution patterns, i.e., regional species pool from published articles, books and flora. These data were well processed in Li et al. [22]. The data of local species pool were from an intensive field survey [23–26]. We aim to test whether regional processes impact the local communities, and if yes, how big the effect is; then we focus on whether the effects are different in different vegetation types, i.e., in grassland and forest.

## Methods

### Study area

**Fig 1:**
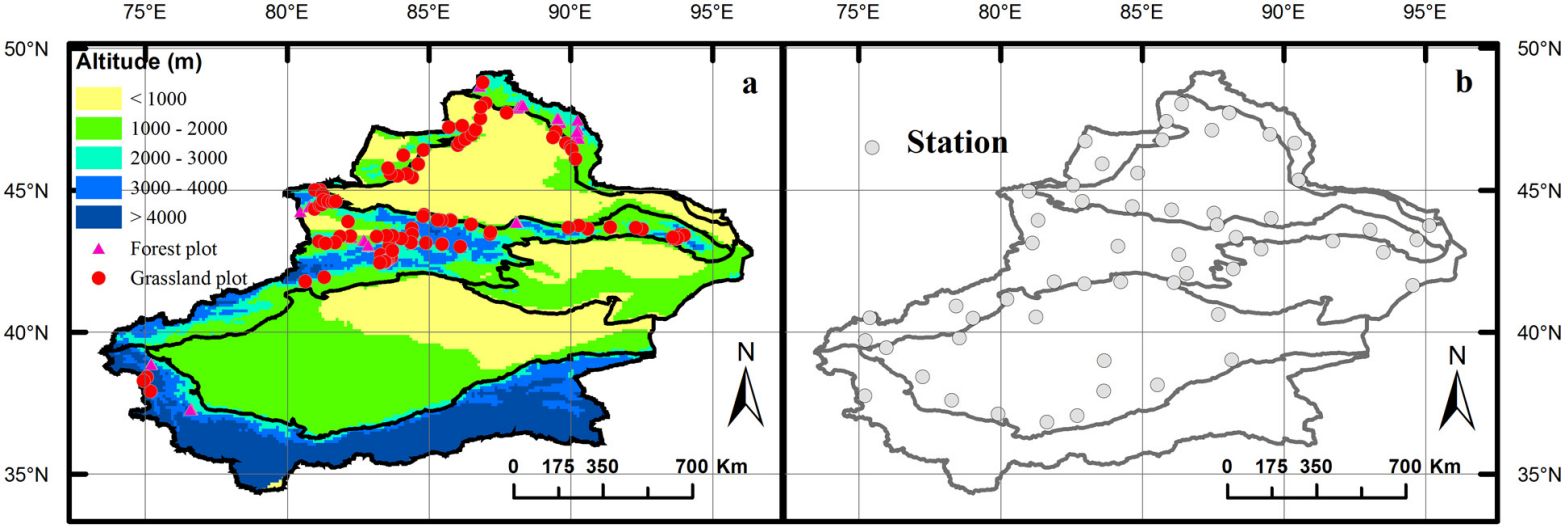
Sampling plots of forest and grassland (a) and the distribution of meteorological stations (b) in Xinjiang. Altitude is shown as the background of (a) and the rough biological divisions as background of both (a) & (b). The data of altitude were downloaded and processed from United States Geological Survey (USGS). The biological division of Xinjiang is shown according to Li et al. [22].

Xinjiang is located in far northwest China with an area of 1.64 million km^**2**^. The geography and climate are various, reaching from high mountains (8611 m above sea level) to low basins (156 m below sea level), from closed forests to open grasslands and from extreme arid to extreme cold climate (see background of Fig 1a for the altitudinal patterns). The vegetation types in Xinjiang are mainly desert and grassland (including steppe and meadow), whereas forest only covers relatively small areas. The dominant species of grassland are from Gramineae, Compositae and Leguminosae. The constructive species of forest are needle trees of *Larix*, *Picea*, *Abies*, and *Pinus*; broadleaved trees of *Betula* and *Populus*; and also native fruit trees of *Malus*, *Armeniaca* and *Juglans* that are endemic to Ily Valley [27].

### Data sources

In this study, RSR was assembled from Li et al. [22]. The dataset is a full collection of all vascular plant species distributions in Xinjiang in a resolution of 0.1 × 0.1 arc degree (with equal area to avoid the area effect). This is, as far as we know, till now the most thorough description of the regional species richness of Xinjiang. The basic data were county level distributions from the record of *Florae Xinjiangensis* [28]. Through the method of GIS, species richness of each grid cell was overlaid and summed. Considering the area of counties in Xinjiang is relatively big and certain species could appear only in restricted environments, also the altitude of species occurrence was considered. We marked the species distributions only when the altitudinal ranges of species overlapped the altitudinal ranges of the grid cells. The regional climate of each grid cell was from the WorldClim Database [29].

The data of LSR (coniferous forest, broadleaved forest and grassland) were mainly collected during 2004–2007 by field survey [23–25]. In addition, 45 plots, mainly broadleaved forests in Ily Valley from Yan and Xu [26] were also included. Totally 190 plots of forest and 103 plots of grassland were collected. The size of a grassland plot was 10 m × 10 m and of a forest plot 20 m × 30 m. Species appeared in each plot and the geographical variables (including latitude, longitude and altitude) of the plot were recorded during the investigation. Attention was paid to ensure that the plots represented characteristic conditions and species composition. Plots of forest vegetation were mostly sampled in nature reserves. Plots of grassland vegetation were sampled in much wider ranges; vegetation types including alpine meadow, alpine steppe, sub-alpine meadow, montane meadow, meadow steppe, temperate steppe, desert steppe and steppe desert were investigated. Climate data of each plot were from the 56 meteorological stations of Xinjiang (Distribution of the stations see Fig 1b). These stations have recorded monthly temperature and precipitation of Xinjiang for about 50 years. The plot climate data, i.e., mean annual temperature (MAT) and precipitation (MAP) were estimated based on latitude (Lat), longitude (Lon) and altitude (Alt) with the method of Wang et al. [30]:

MAP (MAT) = a×Lat + b×Lon + c×Alt

Soil pH of Xinjiang was digitalized from *The Soil Atlas of China* [31]. The soil pH data were resampled into the same resolution as the plant species richness data for the further analysis (see S1 Fig for the distribution patterns of soil pH in Xinjiang region).

### Data analyses

First, we sampled regional species richness according to the latitude and longitude of plots as RSR of plot level. We then averaged the species richness of plots that were located in the same grid cell to get LSR of a grid cell. The plots of forest were more concentrated than the plots of grassland. Totally 32 grid cells of the forest communities and 87 grid cells of the grassland communities were obtained. Second, ordinary least square (OLS) regressions were conducted to analyze the relationship of RSR and soil pH, geographical variables, climate variables. Then, General Linear Models (GLMs) were conducted to find the relative effects of RSR and climate variables on LSR.

The above mentioned linear regressions were usually widely used to detect the relationships of regional and local species richness. However, later studies found that this method may increase the possibility of unsaturated patterns. Alternatively, a new log-ratio-based regression model was proposed that may reflect more the real pattern [18]. Thus, also the log-ratio method was conducted in the analysis for comparing it with the result of linear regressions. The statistical analyses were conducted in R 3.0.3 [32]. The whole dataset, including RSR, LSR and the corresponding climate variables was supplied as S1 File.

## Results

### The geographical distribution patterns of RSR and LSR

**Fig 2:**
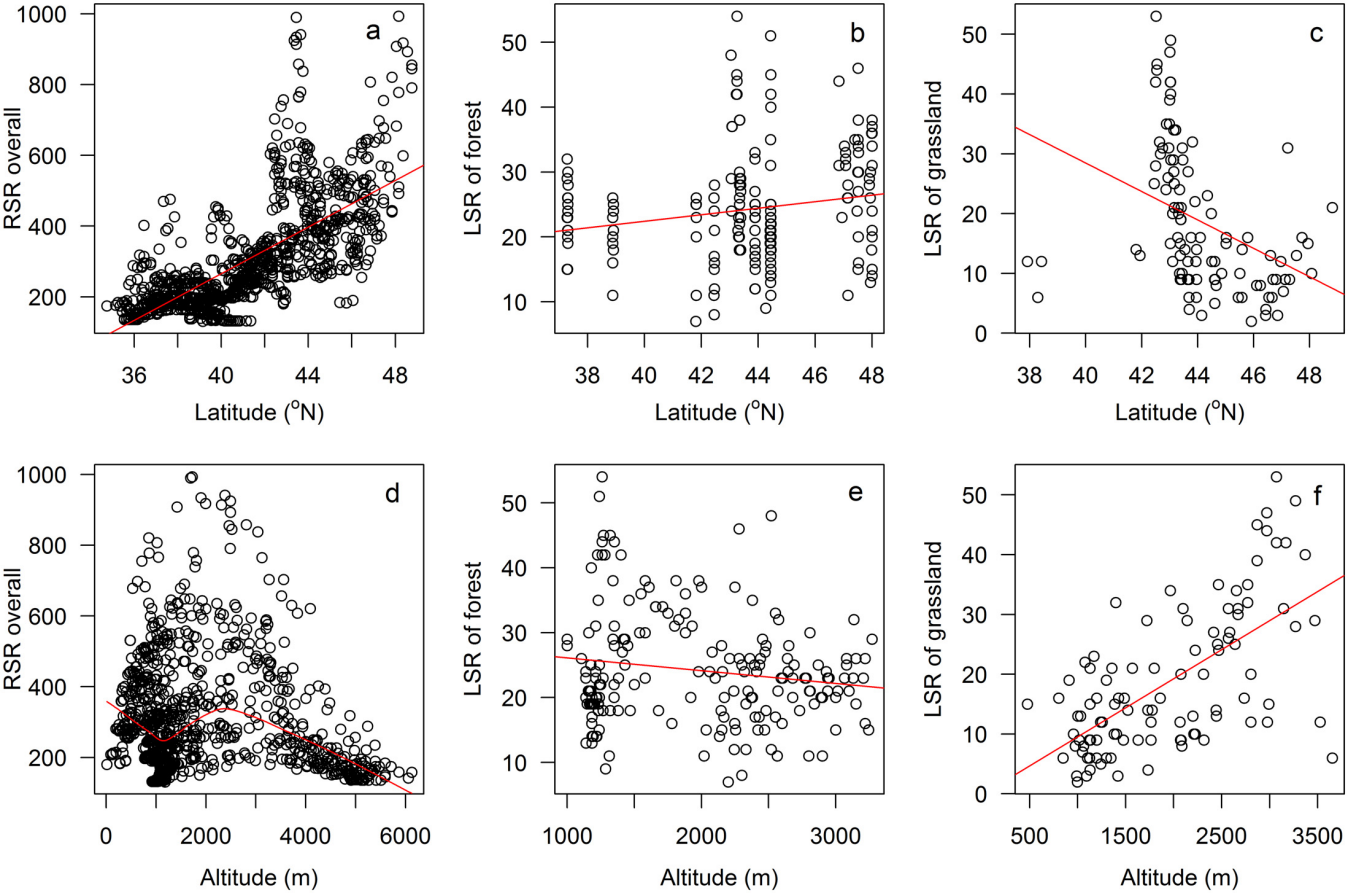
The relationships of regional species richness (RSR), local species richness (LSR) of forest, grassland and latitude, altitude.

**Fig 3:**
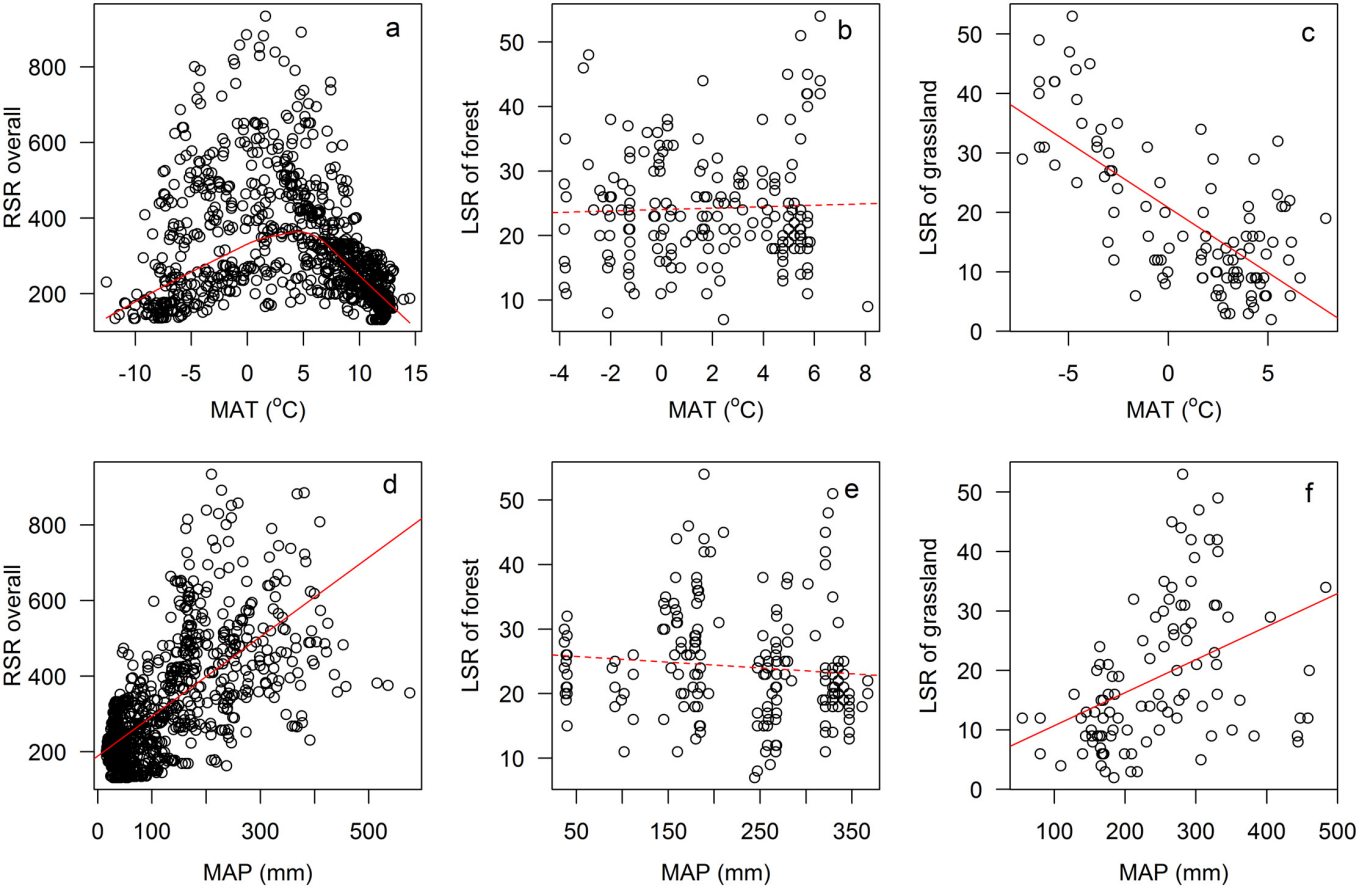
The relationships of regional species richness (RSR), local species richness (LSR) of forest, grassland and MAT (mean annual temperature), MAP (mean annual precipitation).

For the whole dataset of 293 observations, RSR showed a minimum of 186, a maximum of 1082, and an average of 539; and 2, 54 and 22 for LSR, respectively. In the whole Xinjiang region, RSR increased with latitude (*r* ^*2*^ = 0.50, *p* < 0.05, Fig 2a), while decreased, increased and decreased with altitude (*r* ^*2*^ = 0.11, *p* < 0.05, Fig 2d). RSR was quadratically correlated with MAT (*r* ^*2*^ = 0.40, *p* < 0.05, Fig 3a) and increased with MAP (*r* ^*2*^ = 0.44, *p* < 0.05, Fig 3d). LSR of forest slightly increased with latitude and decreased with altitude (*r* ^*2*^ = 0.03 & 0.02, *p* < 0.05, Fig 2b and 2e) and did not change significantly with MAT and MAP (*p* > 0.05, Fig 3b and 3e). LSR of grassland decreased with latitude and MAT (*r* ^*2*^ = 0.14 & 0.49, *p* < 0.05, Figs 2c and 3c) and increased with altitude and MAP (*r* ^*2*^ = 0.40 & 0.17, *p* < 0.05, Figs 2f and 3f).

### The relationship of RSR and soil pH in mountains and basins

**Fig 4:**
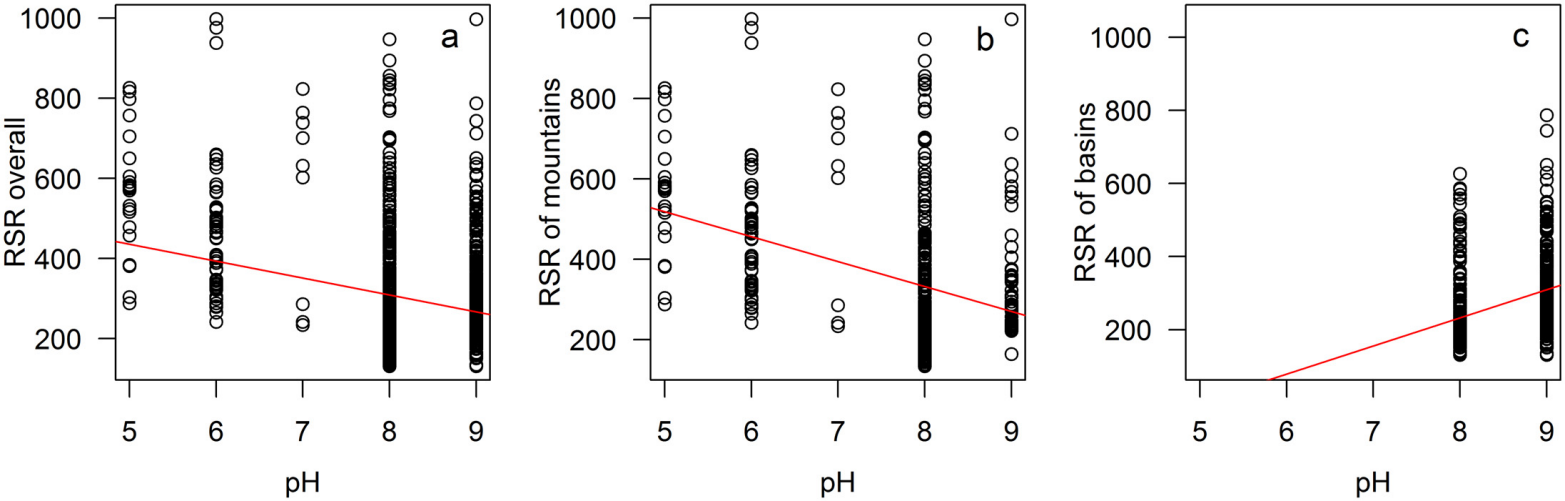
The relationships of RSR and soil pH in the whole Xinjiang region (a), mountain areas (b) and basin areas (c).

In the whole Xinjiang, RSR was negatively correlated with soil pH (*r* ^*2*^ = 0.05, *p* < 0.05, Fig 4a). Particularly, the trends were opposite in mountains and basins. In mountains, RSR was negatively correlated with soil pH, and pH explained 11.2% of the variances of plant species richness (*p* < 0.05, Fig 4b). By contrast, in basins, RSR was positively correlated with soil pH, and pH explained 11.7% of the variances of plant species richness (*p* < 0.05, Fig 4c).

### The relationship of RSR and LSR in forest and grassland communities

**Fig 5:**
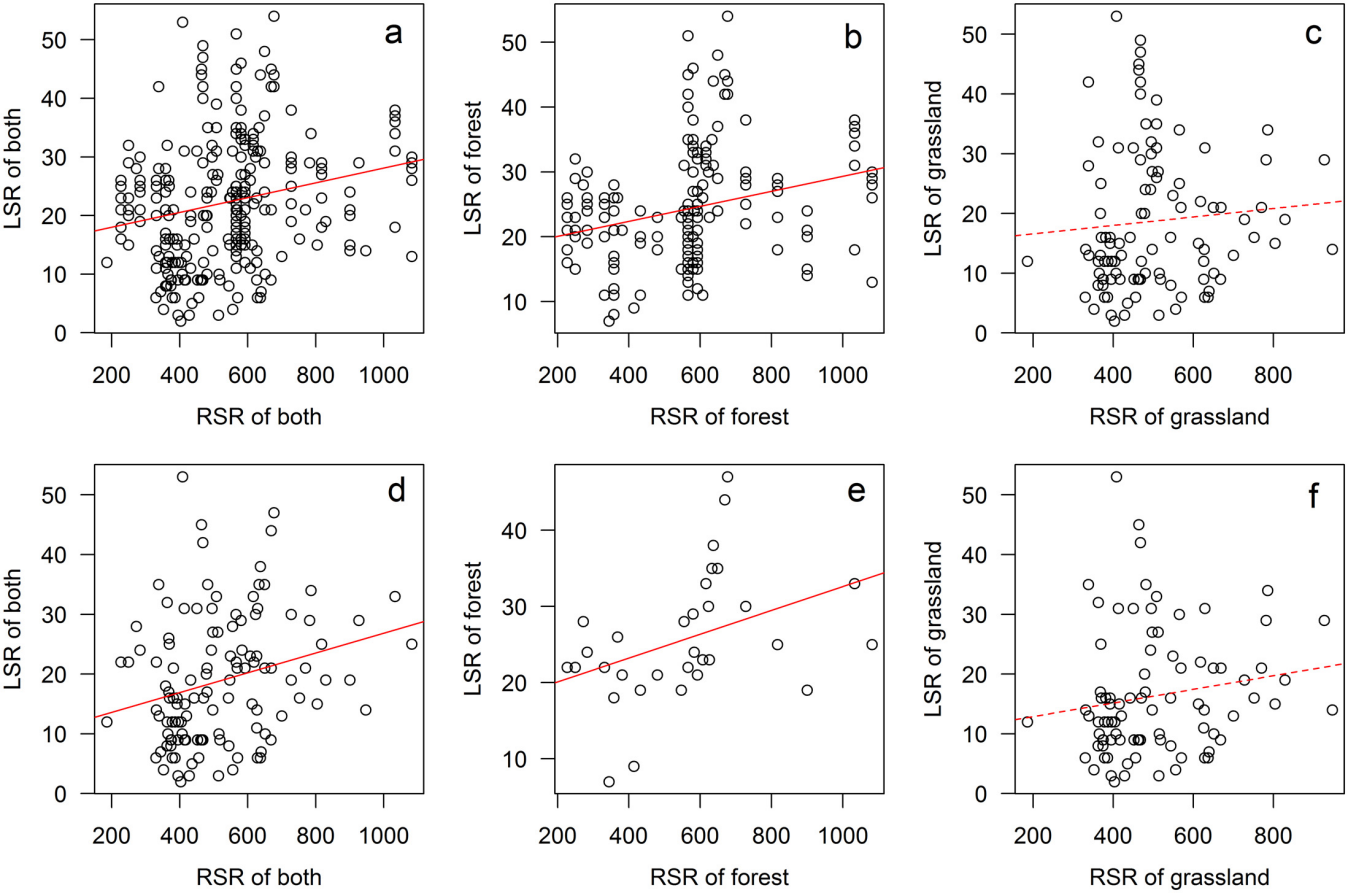
The linear relationships between LSR and RSR for forest and grassland plots pooled together (a & d) and for forest and grassland plots separately (b & e, c & f). Upper row (a, b & c) for plot level data and bottom row (d, e & f) for grid level data; grid level data were the dataset of average species richness of plots that were geographically located in the same grid cell; solid lines represented significant regressions (*p* < 0.05) and dashed lines were non-significant regressions (*p* > 0.05).

There was a significant effect of RSR on LSR (*r* ^*2*^ = 0.05, *p* < 0.05, Fig 5a) and the effect was different for forest and grassland communities. RSR significantly impacted LSR for forest vegetation (*r* ^2^ = 0.07, *p* < 0.05, Fig 5b). However for the grassland, RSR did not impact LSR significantly (*p* > 0.05, Fig 5c). Similar trends were found by employing grid level data, and were shown in Fig 5d, e&f (*r* ^*2*^ = 0.07 & 0.15, *p* < 0.05 for both communities and forest community; *p* > 0.05 for the grassland community).

**Fig 6:**
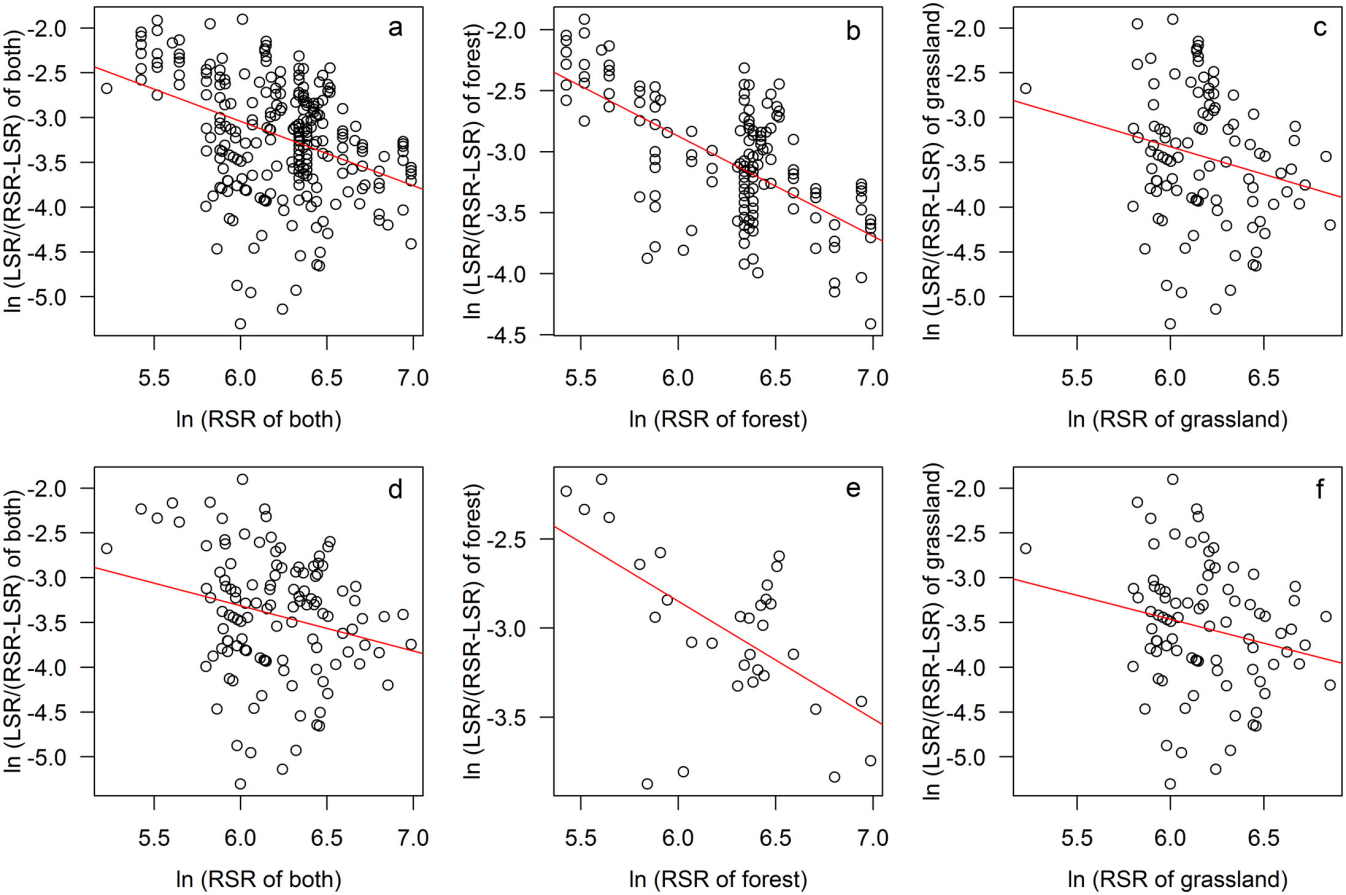
The log-ratio-based relationships between LSR and RSR for forest and grassland plots pooled together (a & d) and for forest and grassland plots separately (b & e, c & f). Upper row (a, b & c) for plot level data and bottom row (d, e & f) for grid level data; grid level data were the dataset of average species richness of plots that were geographically located in the same grid cell.

Furthermore, the results of log-ratio-based regressions confirmed the result of linear regressions. It showed that the slope of both forest and grassland was between 0 and -1 (slope = -0.82±0.07 & -0.61±0.28 for forest and grassland of plot level data, and slope = -0.66±0.17 & -0.53±0.27 for forest and grassland of grid level data, respectively; these values were “intermediate” according to the definition of Szava-Kovats et al. [18] which meant both the relationships were between fully saturated and totally unsaturated). This meant that the LSR of both communities was influenced by RSR. The *r* ^*2*^ of forest was much higher than that of grassland which might reflect that the effect of RSR was much stronger in forest than in grassland (*r* ^*2*^ = 0.43 & 0.05 for forest and grassland of plot level data; *r* ^*2*^ = 0.34 & 0.05 for forest and grassland of grid level data; Fig 6).

### The impact of RSR and climate on LSR in forest and grassland communities

Not only RSR, but also climate variables impacted LSR (*r* ^2^ = 0.17 for MAT, *r* ^2^ = 0.11 for RSR, both *p* < 0.05; *r* ^2^ = 0.02 for MAP, *p* > 0.05). The relative effects of RSR and climate varied for forest and grassland. In the forest community, RSR significantly impacted LSR (*r* ^2^ = 0.20, *p* < 0.05) but climate did not (*p* > 0.05). However in the grassland community, the effect of climate on LSR was much bigger than that of RSR (*r* ^2^ = 0.13 & 0.25 for MAP and MAT, *r* ^2^ = 0.09 for RSR, all *p* < 0.05, Table 1).

**Table 1:** Effect of climate variables and regional species richness (RSR) in explaining local species richness (LSR). For forest and grassland biomes pooled together, and for forest and grassland plots separately.

	Both biomes			Forest			Grassland		
	*df*	*SS%*	*p*	*df*	*SS%*	*p*	*df*	*SS%*	*p*
MAP	1	2.2	0.06	1	0.8	0.57	1	13.0	< 0.05
MAT	1	17.3	< 0.05	1	1.7	0.44	1	24.7	< 0.05
RSR	1	10.8	< 0.05	1	19.5	< 0.05	1	9.0	< 0.05
Residuals	115	69.7		28	78.0		83	53.3	

MAP, mean annual precipitation; MAT, mean annual temperature; df, degree of freedom; SS%, ratio of the sum squares.

## Discussion

### The distribution patterns of RSR and LSR

With the specific mountain—basin system, we found that RSR increased with latitude. The overall species richness was relatively higher in the north (e.g., Altay Mountains and Jungar Basin) than in the south (e.g., Kunlun Mountains and Tarim Basin). LSR of grassland communities correlated more with environmental variables than that of forest communities.

As Xinjiang is located in the inland of Asia that is far from sea, precipitation is scarce for most areas. Plant species richness is determined by not only the amount of energy that it gets but also rainfall it receives, i.e. both potential evapotranspiration (PET) and actual evapotranspiration (AET) [22]. Further, the correlation (estimated by *r* value) of RSR and climate is stronger than that of the LSR and climate for the whole region [22,24]. This may imply that LSR is more affected by local environments, for example, topographical heterogeneity [33], while RSR is more affected by large scale climate.

### The effect of pH on RSR

High species richness in mountains probably was caused by the moderate temperature and heterogeneous environment. On the contrary, the species richness being low in basins was probably caused by the more stressed environment, i.e., extremely arid climate conditions. The different effects of soil pH on RSR in mountains and basins might be caused by the different historical origins of these regions. Pärtel [19] analyzed the relationship between species richness and soil pH with 85 spots distributed worldwide and found that species richness was negatively correlated with pH values in low latitude whereas the relationship was positive in high latitude. This conclusion was widely tested in different regions and vegetation types [20,21]. Comparing our study in Xinjiang with Pärtel’s worldwide analysis [19], we assume that the relationship of RSR and soil pH in mountain habitats of Xinjiang corresponds to low latitude, while the relationship in basins is comparable with high latitude. Soil in basins was in the long history more entisols with high pH values which indicated that species richness could be more possibly high in high soil pH area. On the contrary, soils in mountains were more often alfisols and mollisols with low pH values which indicated possibly high species richness in low pH area.

### The effect of climate and RSR on LSR

The effect of RSR on LSR was already observed for a long time in previous research. The richness of local communities is open to enrichment from the regional species pool [17,34], e.g., a linear relationship of RSR and LSR at the transect-region scale for corals was found even the local scale was very small relative to the regional scale [34]. This shows that regional influences can penetrate very small localities to increase local richness even in extraordinarily rich regions. It was found that filtering of species from the regional species pool into local communities was influenced by local and regional processes, and also evolutionary history; the importance of different filters changed over succession stages [35] or geographic area [15], for example, through a simulation in woody communities across China’s mountains, Wang et al. [15] found that the regional enrichment in temperate regions was more than the combined effects of abiotic filtering and biotic competition in tropical regions.

Through the method of linear regressions and log-ratio-based regressions, we found that LSR was influenced by RSR in the forest of Xinjiang. The results were slightly different for grassland. LSR of grassland was not influenced by RSR with the method of linear regressions (*p* = 0.42 and 0.14 in the plot and grid level, respectively), and it was weakly influenced by RSR with the method of log-ratio-based regression (*p* = 0.03 and 0.05 in the plot and grid level, respectively). Even so, the *r* ^2^ values of grassland were much lower than that of forest. Therefore, we concluded that RSR has stronger effect on LSR in forest than in grassland of Xinjiang region in China.

We found significant effects of both RSR and climate on LSR all over Xinjiang region; however, the relative effects of climate and RSR on LSR were different in forest and grassland. The differences were probably due to the different characters of the two vegetation types. The species richness of forest in Xinjiang was primarily determined by herbaceous plant species under canopy which was influenced more by the small scale environment variables, such as crown density. By contrast, without the shadow of tree species, the species richness of grassland was more influenced by solar radiation, climate, soil, and altitude; but was less influenced by RSR. Zobel et al. [16] stressed that predictions made by the species pool hypothesis were specific to a habitat type and could not be extended to interpretation for diversity at the landscape level where different habitat types co-occurred. Our results confirmed that the relationship of RSR and LSR is different in various vegetation types. Therefore, cautions are needed when doing general analyses in large areas with several habitat types, as complex relationships between LSR and RSR may occur.

## Conclusions

In the forest of Xinjiang, RSR had a more profound role on LSR than climate. An inverse conclusion was found for grassland where climate played a more significant role on LSR while the relative effect of RSR was weaker. Besides this, the effect of soil pH on RSR in mountains was opposite of which in basins in this region. RSR increased with pH values in basins and decreased with pH values in mountains. We concluded that LSR is determined variously in different vegetation types and also different habitats, i.e., the effect from regional species pool on local species pool could be different within the same region. The general analyses might conceal the specific patterns of small scales.

## Supporting Information

S1 Fig The distribution patterns of soil pH in Xinjiang region, China. (TIF)

S1 File The data of RSR, LSR and corresponding climate variables. (XLSX)

## References

[1] Fang JY, Wang XP, Tang ZY. Local and regional processes control species richness of plant communities: The species pool hypothesis. Biodiversity Science 2009;17(6):605–612.

[2] Hawkins BA, Field R, Cornell HV, Currie DJ, Guegan J-F, Kaufman DM, et al Energy, water, and broad-scale geographic patterns of species richness. Ecology 2003;84(12):3105–3117.

[3] Wang ZH, Brown JH, Tang ZY, Fang JY. Temperature dependence, spatial scale, and tree species diversity in eastern Asia and North America. Proceedings of the National Academy of Sciences of the United States of America 2009;106(32):13388–13392. 10.1073/pnas.0905030106 19628692 PMC2714761

[4] Latham RE, Ricklefs RE. Global patterns of tree species richness in moist forests: energy-diversity theory does not account for variation in species richness. Oikos 1993;67(2):325–333.

[5] Parmentier I, Malhi Y, Senterre B, Whittaker RJ, Alonso A, Balinga MP, et al The odd man out? Might climate explain the lower tree α-diversity of African rain forests relative to Amazonian rain forests? Journal of Ecology 2007;95(5):1058–1071.

[6] Laanisto L, Urbas P, Pärtel M. Why does the unimodal species richness–productivity relationship not apply to woody species: a lack of clonality or a legacy of tropical evolutionary history? Global Ecology and Biogeography 2008;17(3):320–326.

[7] Wang XP, Tang ZY, Shen ZH, Zheng CY, Luo JC, Fang JY. Relative influence of regional species richness vs local climate on local species richness in China's forests. Ecography 2012;35(12):1176–1184.

[8] Zobel M. Plant species coexistence: the role of historical, evolutionary and ecological factors. Oikos 1992;65(2):314–320.

[9] Ricklefs RE, Latham RE, Qian H. Global patterns of tree species richness in moist forests: distinguishing ecological influences and historical contingency. Oikos 1999;86(2):369–373.

[10] Zobel M. The relative role of species pools in determining plant species richness: An alternative explanation of species coexistence? Trends in Ecology and Evolution 1997;12(7):266–269. 21238064 10.1016/s0169-5347(97)01096-3

[11] Cornell HV, Lawton JH. Species interactions, local and regional processes, and limits to the richness of ecological communities: a theoretical perspective. Journal of Animal Ecology 1992;61:1–12.

[12] Eriksson O. The species-pool hypothesis and plant community diversity. Oikos 1993;68(2):371–374.

[13] Taylor DR, Aarssen LW, Loehle C. On the relationship between r/K selection and environmental carrying capacity: a new habitat templet for plant life history strategies. Oikos 1990;58(2):239–250.

[14] Zobel M, van der Maarel E, Duprx C. Species pool: The concept, its determination and significance for community restoration. Applied Vegetation Science 1998;1(1):55–66.

[15] Wang SP, Tang ZY, Qiao XJ, Shen ZH, Wang XP, Zheng CY, et al The influence of species pools and local processes on the community structure: a test case with woody plant communities in China's mountains. Ecography 2012;35(12):1168–1175.

[16] Zobel M, Otto R, Laanisto L, Naranjo-Cigala A, Pärtel M, Fernández-Palacios JM. The formation of species pools: historical habitat abundance affects current local diversity. Global Ecology and Biogeography 2011;20(2):251–259.

[17] Harrison S, Cornell H. Toward a better understanding of the regional causes of local community richness. Ecology Letters 2008;11(9):969–979. 10.1111/j.1461-0248.2008.01210.x 18513314

[18] Szava-Kovats RC, Ronk A, Pärtel M. Pattern without bias: local-regional richness relationship revisited. Ecology 2013;94(9):1986–1992. 24279270 10.1890/13-0244.1

[19] Pärtel M. Local plant diversity patterns and evolutionary history at the regional scale. Ecology 2002;83(9):2361–2366.

[20] Chytrý M, Danihelka J, Ermakov N, Hájek M, Hájková P, Kočí M, et al Plant species richness in continental southern Siberia: effects of pH and climate in the context of the species pool hypothesis. Global Ecology and Biogeography 2007;16(5):668–678.

[21] Schuster B, Diekmann M. Changes in species density along the soil pH gradient—evidence from German plant communities. Folia Geobotanica 2003;38(4):367–379.

[22] Li LP, Wang ZH, Zerbe S, Abdusalih N, Tang ZY, Ma M, et al Species richness patterns and Water-Energy Dynamics in the drylands of northwest China. PLoS ONE 2013;8(6):e66450 10.1371/journal.pone.0066450 23840472 PMC3688736

[23] Li LP, Hai Y, Mohammat A, Tang ZY, Fang JY. Community structure and conservation of wild fruit forests in the Ili Valley, Xinjiang. Arid Zone Research 2011;28(1):60–66.

[24] Li LP, Mohammat A, Guo ZD, Hai Y, Tang ZY. Study on plant species composition and richness of the mountain coniferous forests in Xinjiang. Arid Zone Research 2011;28(1):40–46.

[25] Mohammat A. Carbon and Nitrogen Storage of Grassland Ecosystem in Xinjiang [PhD Thesis]. Beijing: Peking University; 2006.

[26] Yan GR, Xu Z. Wild fruit tree resources in Tianshan Mountains. Northern Horticulture 2001;(1):24–27.

[27] Xinjiang Integrated Survey Team, Institute of Botany, Chinese Academy of Sciences. The Vegetation and Its Utilization in Xinjiang. Beijing: Science Press; 1978.

[28] Commissione Redactorum Florae Xinjiangensis. Florae Xinjiangensis. Urumqi: Xinjiang Science & Technology Publishing House; 1992–2011.

[29] Hijmans RJ, Cameron SE, Parra JL, Jones PG, Jarvis A. Very high resolution interpolated climate surfaces for global land areas. International Journal of Climatology 2005;25:1965–1978.

[30] Wang XP, Fang JY, Tang ZY, Zhu B. Climatic control of primary forest structure and DBH-height allometry in Northeast China. Forest Ecology and Management 2006;234(1–3):264–274.

[31] Institute of Soil Science, Chinese Academy of Sciences. The Soil Atlas of China. 1st ed Beijing: Cartographic Publishing House; 1986.

[32] R Development Core Team. R: a language and environment for statistical computing. 3.0.3 ed Vienna, Austria: R Foundation for Statistical Computing; 2014.

[33] Zelený D, Li CF, Chytrý M. Pattern of local plant species richness along a gradient of landscape topographical heterogeneity: result of spatial mass effect or environmental shift? Ecography 2010;33(3):578–589.

[34] Cornell HV, Karlson RH, Hughes TP. Local-regional species richness relationships are linear at very small to large scales in west-central Pacific corals. Coral Reefs 2008;27(1):145–151.

[35] Marteinsdóttir B, Eriksson O. Trait-based filtering from the regional species pool into local grassland communities. Journal of Plant Ecology 2014;7(4):347–355.

