# Supplementary figures and images for: Different Effects of Regional Species Pool on Plant Diversity between Forest and Grassland Biomes in Arid Northwest China
Source: PLoS One. 2015 Jul 2;10(7):e0131982. doi: 10.1371/journal.pone.0131982 (PMC4489744; doi:10.1371/journal.pone.0131982)

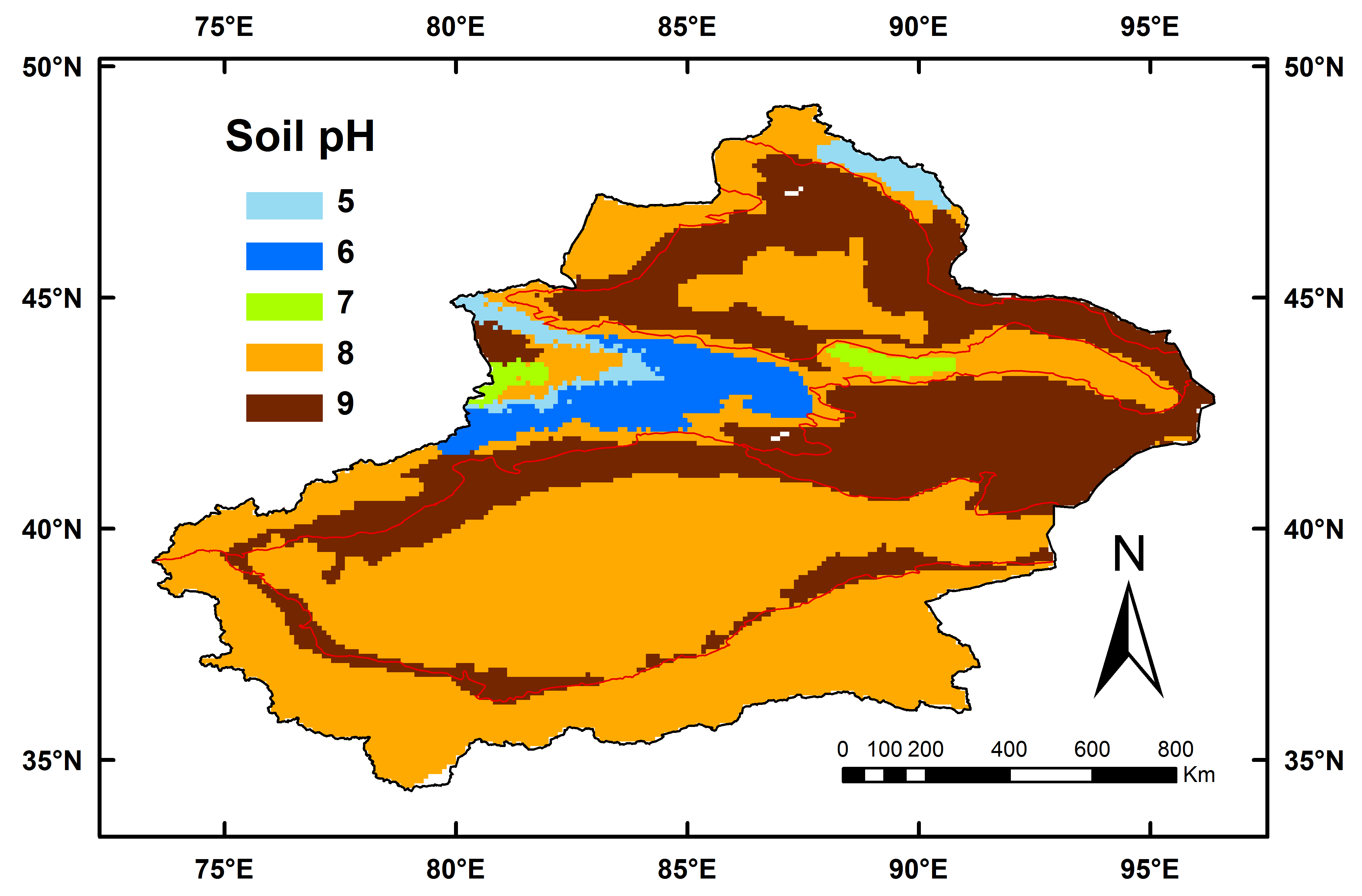

Supplement: S1 Fig — (TIF) [file pone.0131982.s001.tif]
